# Metagenomic Analysis of Fungal Diversity on Strawberry Plants and the Effect of Management Practices on the Fungal Community Structure of Aerial Organs

**DOI:** 10.1371/journal.pone.0160470

**Published:** 2016-08-04

**Authors:** Ahmed Abdelfattah, Michael Wisniewski, Maria Giulia Li Destri Nicosia, Santa Olga Cacciola, Leonardo Schena

**Affiliations:** 1 Dipartimento di Agraria, Università Mediterranea di Reggio Calabria, Località Feo di Vito, Reggio Calabria, 89122, Italy; 2 USDA-ARS-AFRS, 2217 Wiltshire Road, Kearneysville, West Virginia, United States of America; 3 Dipartimento di Agricoltura, Alimentazione e Ambiente, Università degli Studi, Via S. Sofia 100, 95123 Catania, Italy; The National Orchid Conservation Center of China; The Orchid Conservation & Research Center of Shenzhen, CHINA

## Abstract

An amplicon metagenomic approach based on the ITS2 region of fungal rDNA was used to identify the composition of fungal communities associated with different strawberry organs (leaves, flowers, immature and mature fruits), grown on a farm using management practices that entailed the routine use of various chemical pesticides. ITS2 sequences clustered into 316 OTUs and Ascomycota was the dominant phyla (95.6%) followed by Basidiomycota (3.9%). Strawberry plants supported a high diversity of microbial organisms, but two genera, *Botrytis* and *Cladosporium*, were the most abundant, representing 70–99% of the relative abundance (RA) of all detected sequences. According to alpha and beta diversity analyses, strawberry organs displayed significantly different fungal communities with leaves having the most diverse fungal community, followed by flowers, and fruit. The interruption of chemical treatments for one month resulted in a significant modification in the structure of the fungal community of leaves and flowers while immature and mature fruit were not significantly affected. Several plant pathogens of other plant species, that would not be intuitively expected to be present on strawberry plants such as *Erysiphe*, were detected, while some common strawberry pathogens, such as *Rhizoctonia*, were less evident or absent.

## Introduction

Strawberry (*Fragaria* x *ananassa*) is an important horticultural crop worldwide, produced both conventionally and organically in open fields, greenhouses, and in plastic covered tunnels. Global production of strawberry fruit reached 739,622.443 tons in 2013 [1]. Strawberries, however, are susceptible to several fungal pathogens and thus the use of fungicide treatments is needed to prevent economic losses. A major disease of strawberry is grey mold, caused by *Botrytis cinerea* although, several other fungal pathogens can also infect strawberries and cause diseases that require specific chemical control measures [2–4]. They include *Podosphaera aphanis* (powdery mildew), *Colletotrichum acutatum* (anthracnose), *Phytophthora cactorum* (leather rot, crown rot), *Rhizoctonia* spp. (black root rot, hard brown rot, Rhizoctonia bud and crown rot, leaf blight, web blight, and fruit rot), *Phytophthora fragariae var*. *fragariae* (red stele root rot), *Verticillium dahliae* and *Verticillium albo-atrum* (Verticillium wilt).

Although studies of the strawberry fungal or bacterial community have been conducted [5,6], these reports were either based on isolation/culture techniques and/or did not study the full complement of strawberry aerial organs, (leaves, flowers and fruit) at the same time. Furthermore, these studies did not investigate the impact of conventional chemical treatments on fungal diversity and community structure. Next generation sequencing (NGS), together with the evolution of bioinformatic tools, and the emergence of metagenomic approaches have made it easier to comprehensively analyze microbial communities on or in any type of matrix, including plant tissues. In recent years researchers have widely applied these new technologies in relation to plant pathology and plant microbial ecology studies [6–12]. One of the main advantages of metagenomic approaches over culture-dependent methods, is the ability to theoretically detect all organisms that possess the targeted barcode gene. This includes, organisms that cannot or are extremely difficult to culture, which represent about 99% of the total estimated microbial diversity [13], as well as rare taxa that are usually missed by culturing techniques [14].

The internal transcribed spacer (ITS) regions of ribosomal DNA (rDNA) are the most predominant DNA barcode sequences used for fungal metabarcoding. These regions can be easily amplified and sequenced with universal primers and the corresponding ITS sequence data is highly represented in GenBank and other databases [15,16]. The choice of using either ITS1 or ITS2 is optional since these regions share many properties, and enable similar levels of discrimination [17]. However, ITS2 is generally used because it is less variable in length, lacks the problem of co-amplification of a 5`SSU intron, and is better represented in databases than ITS1 sequences [18].

The objective of the present study was to identify the composition of the fungal communities associated with different strawberry organs and determine the effect of interrupting the application of chemical treatments in a conventional production system. The identification and quantification of endophytic and epiphytic microflora present in and on plants provides information that can facilitate an understanding of the complex interactions that take place between plants and resident fungal microflora, including pathogenic and beneficial species. Such data may also have potential practical implications for strawberry disease management, particularly of economically important pathogens, like *B*. *cinerea*, which can impact fruit yield and quality at both pre-and post-harvest stages of production. Documenting the relative abundance (RA) of fungal pathogens on different plant organs can also greatly assist in understanding the etiology and control of complex plant diseases.

## Materials and Methods

### Ethics statement

The study was carried out on private land and the owner of the land gave permission to conduct the study on this site. The study did not involve endangered or protected species.

### Sampling and DNA extractions

Samples were collected on April 30, 2014 from strawberry field planted in August, 2012 on a farm located in Lamezia Terme, Southern Italy (GPS coordinates: 38°49'37.6"N 16°14'23.0"E) as summarized in Table 1. The strawberry cultivar ‘Rania’ (Consorzio Italiano Vivaisti, Italy) was grown on raised beds under high tunnels. Samples were collected from 6 different rows, 50 m in length, based on a complete randomized block design. Three of the strawberry rows were subjected to regular schedules of chemical treatments during the entire production cycle and included the use of ciprodinil and fludioxonil (Switch, Syngenta, Milan, Italy) on April 1, 2014, pyraclostrobin and Boscalid (Signum, BASF, Cesano Maderno, Italy) on April 10, 2014, and fenexamid (Teldor, Bayer CropScience, Milan, Italy) on April 20, 2014. The other three rows were located in the same field but did not receive any chemical treatments during the month of April. In order to avoid the potential effect of fungicide drift, the untreated rows of strawberry were separated from the treated strawberry rows by two rows. Three biological replicate samples were collected from four different plant organs (leaves, flowers, immature fruit and mature fruit) in each row, resulting in a total of 24 samples. Each biological replicate consisted of a pool of ten leaves, flowers, or immature or mature fruit, collected from ten individual plants. Collected samples were kept in sterile plastic bags in a thermally insulated container for approximately 4–5 hours, lyophilized using a freeze dryer (Labconco Corp., Kansas City, MO) and stored at -80°C until the samples were homogenized by grinding under liquid nitrogen. For each sample, three technical replicate DNA extractions were performed from 0.02 g of homogenized tissue using the DNeasy Plant Mini kit (QIAGEN, Dusseldorf, Germany). The quantity and quality of extracted DNA were determined using a Nanodrop 2000 spectrophotometer (Nano-drop Technologies, Wilmington, DE).

**Table 1:** Summary of analyses and results of metagenomic surveys conducted with treated (T) and untreated (U) leaves (TL and UL), flowers (TFl and UFl), immature fruit (TImFr and UImFr), and mature fruit (TMFr and UMFr).

Samples	MIDs^a^	Sequences	OTUs (Total)^b^	OTUs (200)^c^	Coverage^d^	Shannon diversity	Chao1 estimate
TL	MID21,22,24	2958	83	20.2	0.9888	1.94	47.26
UL	MID36,37,38	3886	118	29.5	0.9876	2.83	64.35
TFl	MID1,2,3	6268	71	12.1	0.9954	1.53	23.17
UFl	MID26,28,40	8207	129	15.8	0.9929	2.1	46.48
TImFr	MID4,6,13	53268	129	10.6	0.9991	1.84	21.09
UImFr	MID30,31,32	42145	109	7.6	0.9989	1.25	10.41
TMFr	MID7,10,16	85879	112	7.9	0.9997	1.29	10.83
UMFr	MID33,34,35	15553	75	7.9	0.9976	1.3	11.1

^a^Multiplex Identifiers;

^b^Total number of detected OTUs;

^c^Number of OTUS detected with an even sequencing depth of 200 sequences;

^d^Estimated sample coverage (Good’s coverage).

### Fungal DNA amplification and amplicon library preparation

DNA extracts from all technical replicates were amplified in triplicate using the universal fungal primers ITS3-ITS4 targeting the ITS2 region of ribosomal DNA [19]. Both primers were modified to construct fusion primers appropriate for 454 sequencing with adapters sequences A and B, key sequences and multiplex identifiers (MIDs) (http://www.454.com/). Twenty-four different MIDs were used to label each biological replicate (Table 1).

PCR reactions were conducted in a total volume of 25 μl containing 2.5 μl of 10X reaction buffer, 0.25 μl of each primer (10μM), 0.1 μl of AccuPrime *Taq* DNA Polymerase High Fidelity (Invitrogen, CA, USA) and 1μl of DNA template (10 ng/μl). Nuclease-free water (QIAGEN, Valencia, CA, USA) replaced template DNA in negative controls. Reactions were incubated in an Eppendorf Mastercycler gradient thermocycler, (Hamburg, Germany) for 1 min at 94°C followed by 30 cycles of 30s at 94°C, 30s at 55°C, and 30s at 68°C. All reactions ended with a final extension of 1 min at 72°C. Amplicons from each biological replicate (3 amplifications for each of the three DNA extractions) were pooled and purified using the Agencourt AMPure XP system (Beckman Coulter, Inc.). The concentration and quality of the purified amplicons was evaluated by agarose gel electrophoresis [20]. Amplicons were sequenced by Macrogen Inc. (Seoul, Korea) using the 454 GS FLX+System (Roche Diagnostics Corporation). Data were deposited in the BioProject database (NCBI) as PRJNA289287 (http://www.ncbi.nlm.nih.gov/bioproject/289287).

### Data processing and analysis

The bioinformatics pipeline, QIIME v. 1.8 [21] was used to process and analyze the obtained sequence data. Using default parameters, preliminary processing of the data included de-multiplexing and quality filtering with a minimum quality score of 25, a minimum/maximum length ratio of 200/1000, and a maximum number of homopolymer bases of 6. Sequences were denoised using the denoise wrapper [22] and the ITS2 region was extracted using ITSx application [23]. Chimeric sequences were identified and filtered using USEARCH 6.1 [24]. Sequences were clustered into Operational Taxonomical Units (OTUs) using the USEARCH 6.1 software and a 97% similarity threshold. The most abundant sequences in each OTU were selected as representative sequences. These sequences were then used for the taxonomy assignments using BLAST [25] and the UNITE dynamic database released on 10.09.2014 (http://unite.ut.ee/) as a reference database.

For downstream analysis, the OTU table was rarefied at an even depth to reduce biases in sequencing depth. Alpha diversity was calculated using observed species, Shannon, Good’s coverage and Chao1 estimates. The results were compared using a nonparametric two-sample t-test and the *P* values were calculated through 999 Monte Carlo permutations. The results were visualized in boxplots figures.

Weighted and unweighted UniFrac metrics were used to evaluate β-diversity [26]. A distance-based redundancy analysis (db-RDA) and Principal coordinates analysis (PCoA) as implemented in Qiime 1.8 was used to relate the fungal community composition to sample types and to evaluate the effect of sample type (plant organ) and treatment (fungicide applications vs. no fungicide applications). The statistical significance of detected differences was evaluated using weighted and unweighted UniFrac distance matrices and 999 Monte Carlo permutations.

### Identification of fungal taxa

To confirm the accuracy of QIIME taxonomic assignments, sequences associated with each OTU within each identified fungal genus with a minimum RA of 0.1% were extracted and compared with validated barcode sequences. Extracted sequences were introduced in ElimDupes (http://hcv.lanl.gov/content/sequence/ELIMDUPES/elimdupes.html) to detect identical sequences and determine their frequency. Unique representative sequences, defined as sequence types (STs) [9,27], were analyzed along with genetically closely-related reference sequences of the same genus to determine their phylogenetic annotation and enable their identification with the highest possible level of accuracy. To this aim, local databases of validated reference sequences were created with priority given to sequences from specific recent taxonomic studies (Annex 1). In cases where no matches were found in the reference sequence from selected published articles (Annex 1) more closely related sequences were selected using BLAST searches of GenBank. The reliability of the latter sequences was evaluated based on different parameters, including the consistency of sequences from different sources, available details on sequenced isolates, and year of publication, giving priority to more recent items. Identical reference sequences were included in the local database when representative of different accepted species.

For each genus, STs identified in the present study, and reference sequences, were aligned using MUSCLE and introduced into MEGA for phylogenetic analysis utilizing the Maximum Likelihood method [28]. Analyses were performed with 1000 bootstrap replications.

## Results

### Alpha diversity and richness

**Fig 1:**
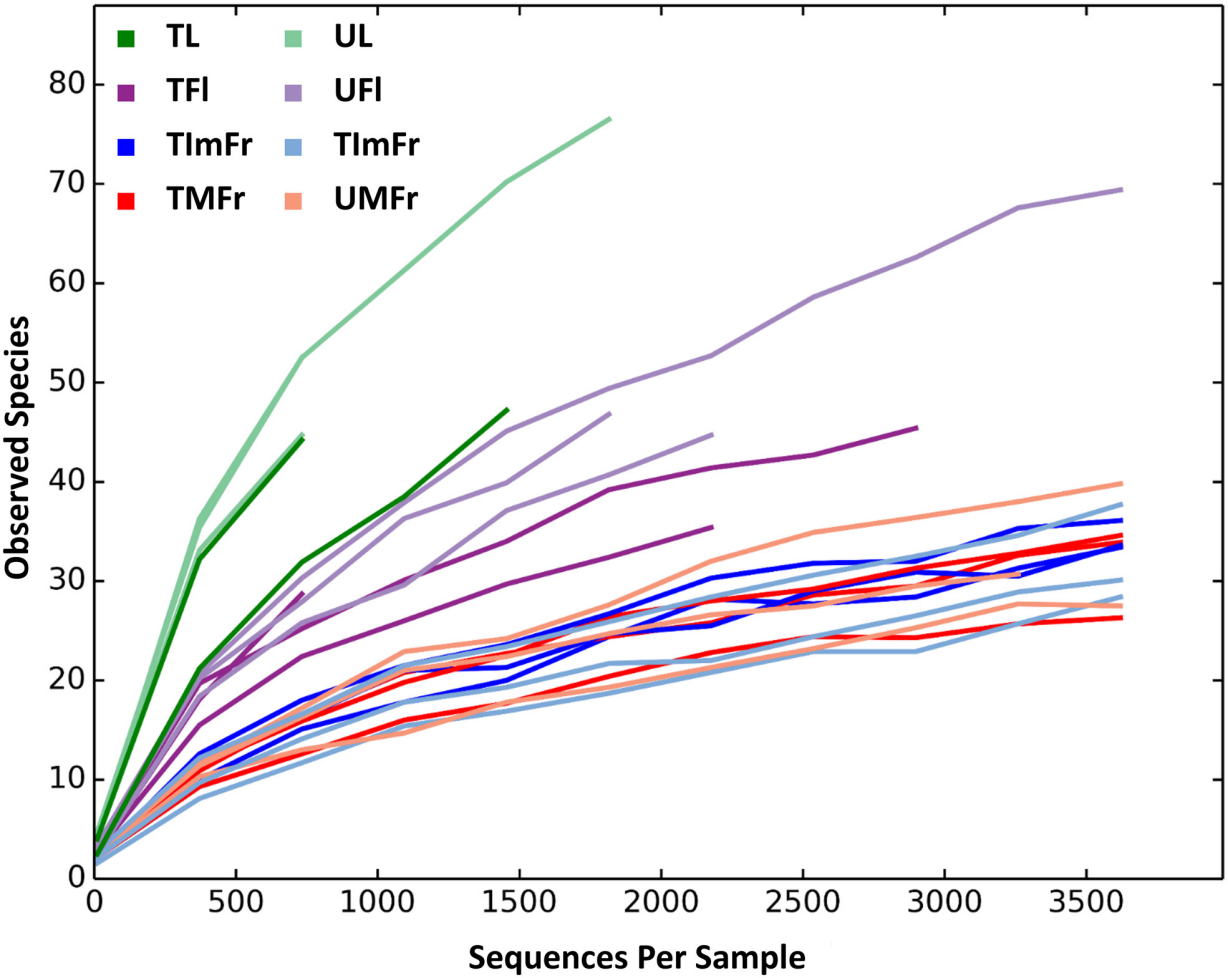
Rarefaction curves determined for all strawberry samples investigated in the present study. Investigated samples included treated (T) and untreated (U) flowers (TFl and UFl), immature fruit (TImFr and UImFr), leaves (TL and UL), and mature fruit (TMFr and UMFr).

After quality evaluations, a total of 218,164 high quality sequences were recovered and assigned to 316 OTUs (Table 1). Sequenced negative control did not yield any fungal sequence. The rarefaction analysis indicated that the sequencing depth had been saturated for most of the samples (Fig 1).

**Fig 2:**
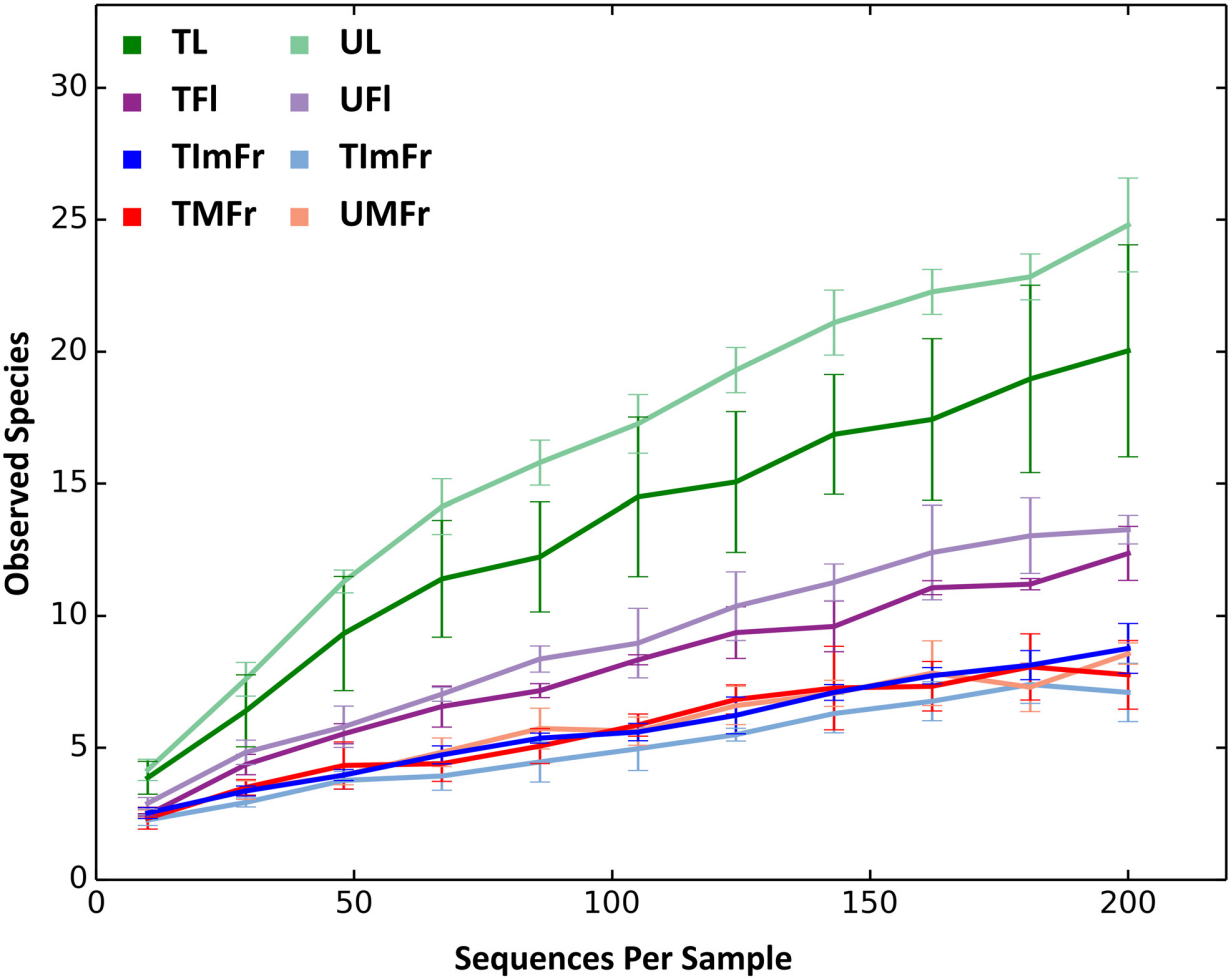
Rarefaction curves of treated (T) and untreated (U) strawberry organs at an even depth of 200 sequences. Samples comprised flowers (TFl and UFl), immature fruit (TImFr and UImFr), leaves (TL and UL), and mature fruit (TMFr and UMFr). Rarefaction curves were grouped to collapse the 3 biological replicates of each analyzed sample type.

**Fig 3:**
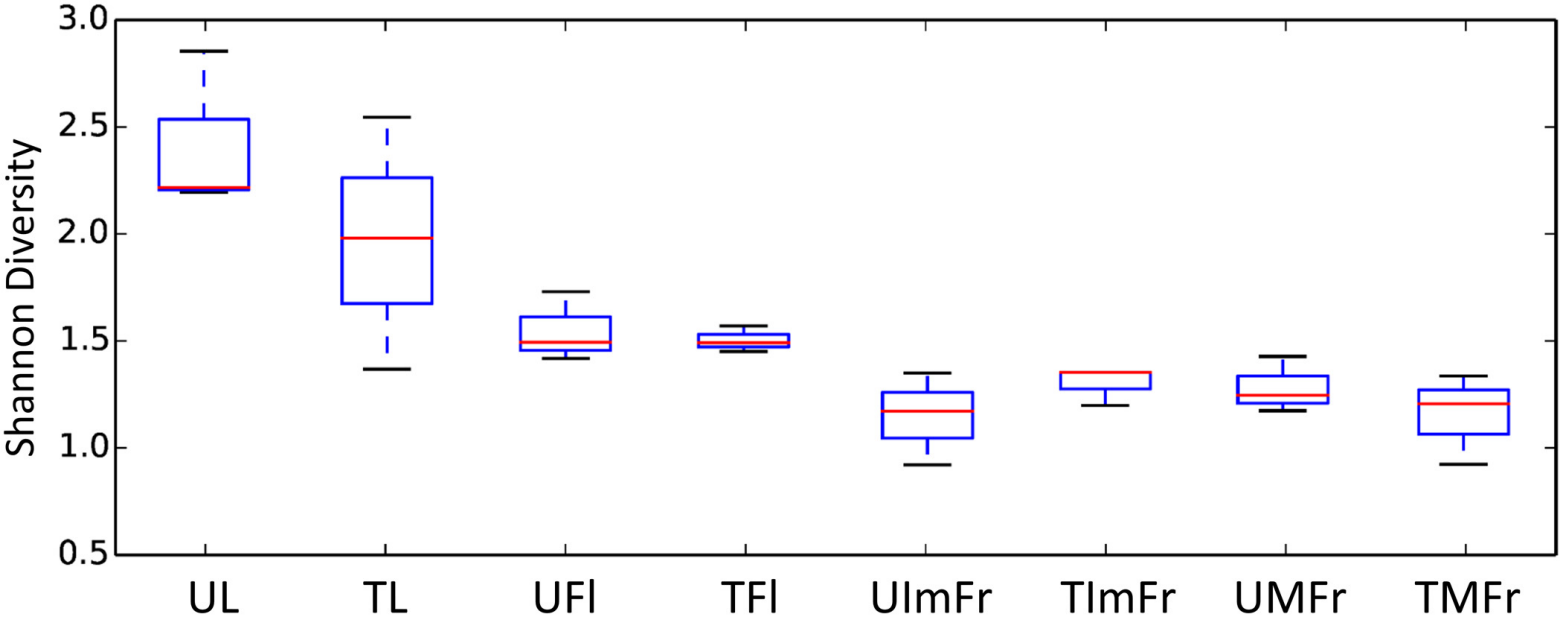
Boxplots visualizing results of the nonparametric two sample t-test to compare the alpha diversity of treated and untreated flowers (TFl and UFl), immature fruit (TIMFr and UIMFr), leaves (TL and UL), and mature fruit (TMFr and UMFr).

The total number of OTUs detected in individual samples varied from 71 to 129. The use of a rarefied OTU table indicated that leaves samples had the highest number of OTUs relative to other organs, followed by flowers, and immature and mature fruit (Table 1). In agreement with the OTU data, the Shannon’s Diversity Index and the Chao1 estimate also revealed that leaves had the greatest level of fungal diversity followed by flowers and fruit. Immature and mature fruit had similar diversity values (Table 1; Fig 2). These results were confirmed by a two-sample t-test with significant differences observed between strawberry organs, except between mature and immature fruit (Fig 3 & Table 2).

**Table 2:** Comparison of alpha diversity between strawberry organs based on Shannon index. P values were determined using 999 Monte Carlo permutations and were considered significantly different when P was ≤ 0.05.

	Leaves	Flowers	Immature fruits	Mature fruits
Leaves	-			
Flowers	0.024	-		
Immature fruits	0.030	0.030	-	
Mature fruits	0.006	0.006	1	-

The interruption of fungicide applications to the strawberry plants increased alpha diversity on leaves and, to a lesser extent, flowers relative to treated samples, although the differences were not statistically significant (Figs 2 and 3). On the other hand, the fungicide applications did not seem to have any noticeable effect on the fungal composition of immature and mature fruit (Figs 2 and 3).

### Beta diversity analysis

**Fig 4:**
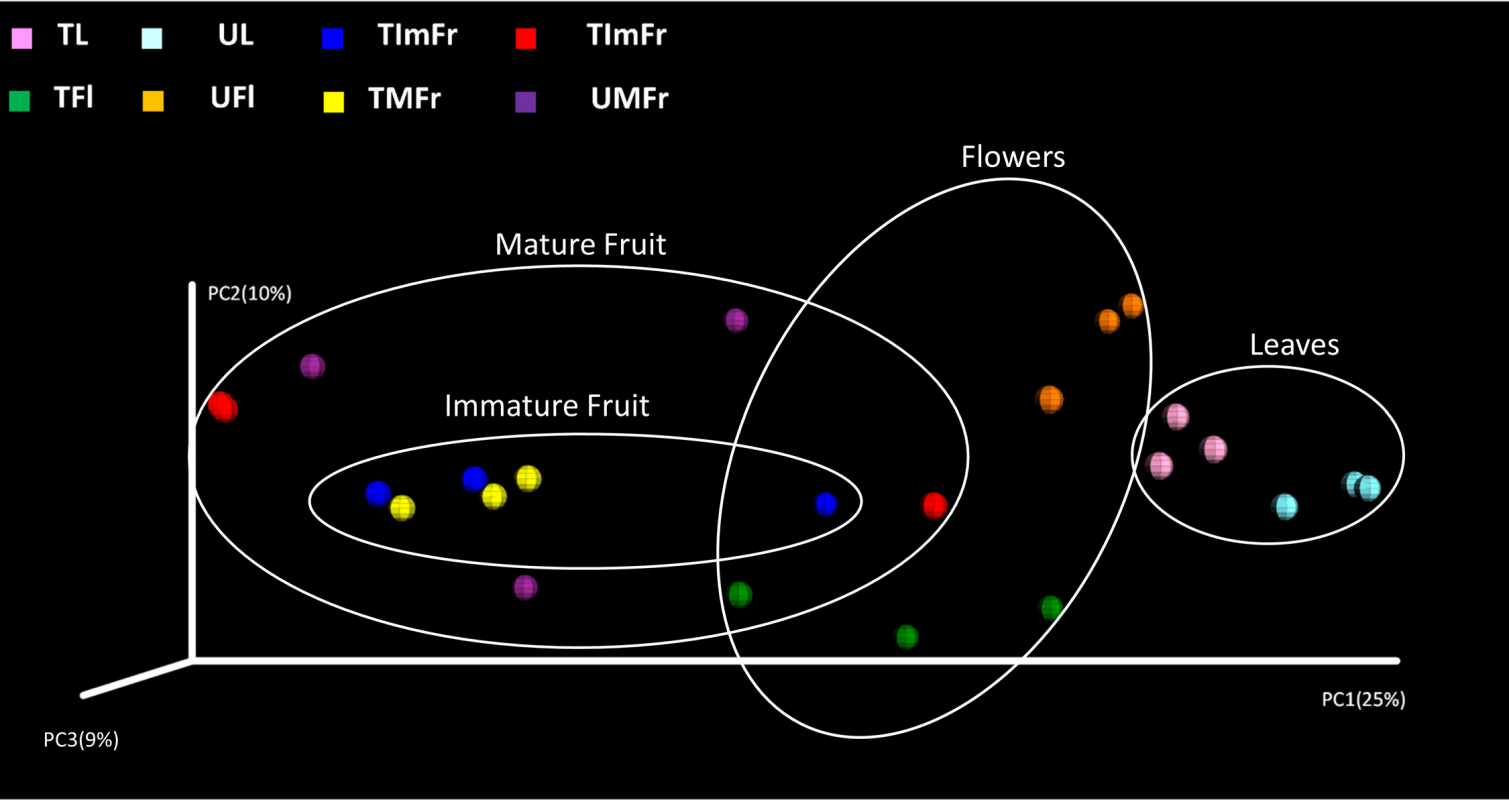
PCoA plot showing the results of the beta diversity analysis using the unweighted UniFrac metrics. White circles were used to indicate samples collected from the same organ, while colored spheres were used to differentiate treated (T) and untreated (U) samples of flowers (TFl and UFl), immature fruit (TIMFr and UIMFr), leaves (TL and UL), and mature fruit (TMFr and UMFr).

The PCoA analysis (Fig 4) as well as the distance-based redundancy test (db-RDA) based on unweighted UniFrac metrics revealed significant differences (*P* < 0.01) between the fungal communities present on all of the investigated strawberry organs (Table 3). Furthermore, significant differences between treated and untreated samples were revealed for leaves (*P* = 0.002) and flowers (*P* = 0.016), while no significant differences were observed between treated and untreated samples for immature and mature fruit (Table 3). However, using the weighted UniFrac metrics a significant difference (*P =* 0.039) was also observed between treated and untreated mature fruit (data not shown).

**Table 3:** Comparison between treated and untreated strawberry organs according to unweighted UniFrac metrics (β-diversity). The significance was determined through 999 Monte Carlo permutations using the distance-based redundancy analysis (db-RDA). Sample groups were considered significantly different if the P value was ≤ 0.05.

Compared categories	Pseudo-F value*	p-value
All categories	1.8353	0.001
All organs	2.2950	0.001
Treated vs. untreated leaves	1.7915	0.002
Treated vs. untreated flowers	1.6419	0.016
Treated vs. untreated mature fruit	1.2647	0.136
Treated vs. untreated immature fruit	1.1818	0.217
Flower vs. mature fruit	1.8448	0.012
Flower vs. leaves	2.0681	0.001
Flower vs. immature fruit	2.2100	0.012
Mature fruit vs. leaves	1.6489	0.011
Mature fruit vs. immature fruit	1.7326	0.003
Immature fruit vs. leaves	1.3996	0.049

*Pseudo-F value determined according to Lattin et al. [29]

### Strawberry fungal community structure and identified taxa

**Fig 5:**
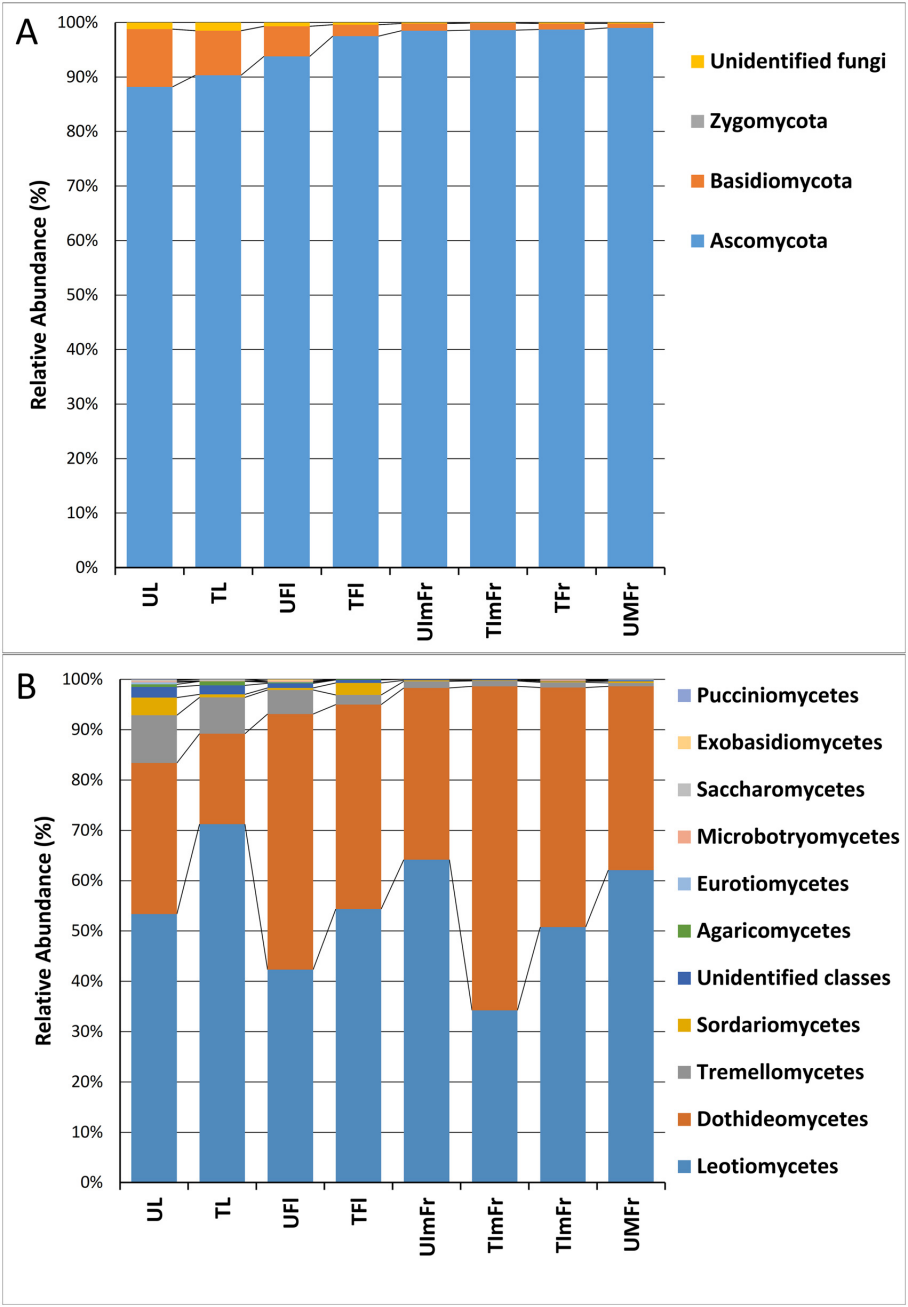
Relative abundance of different fungal phyla (A) and classes (B). Analyzed samples comprised treated (T) and untreated (U) flowers (TFl and UFl), immature fruit (TIMFr and UIMFr), leaves (TL and UL), and mature fruit (TMFr and UMFr).

Based on the analysis of the complete ITS2 data set, members of the phylum *Ascomycota* were dominant in all samples, collectively accounting for 95.6% of the total number of detected sequences, followed by *Basidiomycota* (3.9%) and unidentified fungi (0.3%). The incidence of *Ascomycota* varied in different samples ranging from 88.2% in untreated leaves to 99% in untreated fruit (Fig 5A). Similar values were obtained for treated samples (Fig 5A). *Basidiomycota* accounted for 8.2% and 10.6% of the detected sequences in untreated and treated leaves, respectively, but were much less abundant in other organs (immature and mature fruit). Sequences ascribed to non-identified fungi had similar levels of abundance in all samples (organs).

Ascomycota sequences were largely identified as members of the classes Leotiomycetes and Dothideomycetes (54.0% and 40.2%, respectively), followed by Sordariomycetes (1%). Within Basidiomycota, Tremellomycetes was the most abundant class with an RA of 3.40% (Fig 5B). Leotiomycetes were more abundant on leaves (61%) than on fruit (38.5%). In contrast, members of the class Dothideomycetes were more abundant on fruit (60%) than on leaves (38.5%). Tremellomycetes was the predominant class of Basidiomycota in all of the analyzed samples. Members of this class were more highly represented on leaves and flowers (8.5 and 3.5%, respectively) than on immature and mature fruit (1.2 and 1%, respectively).

**Fig 6:**
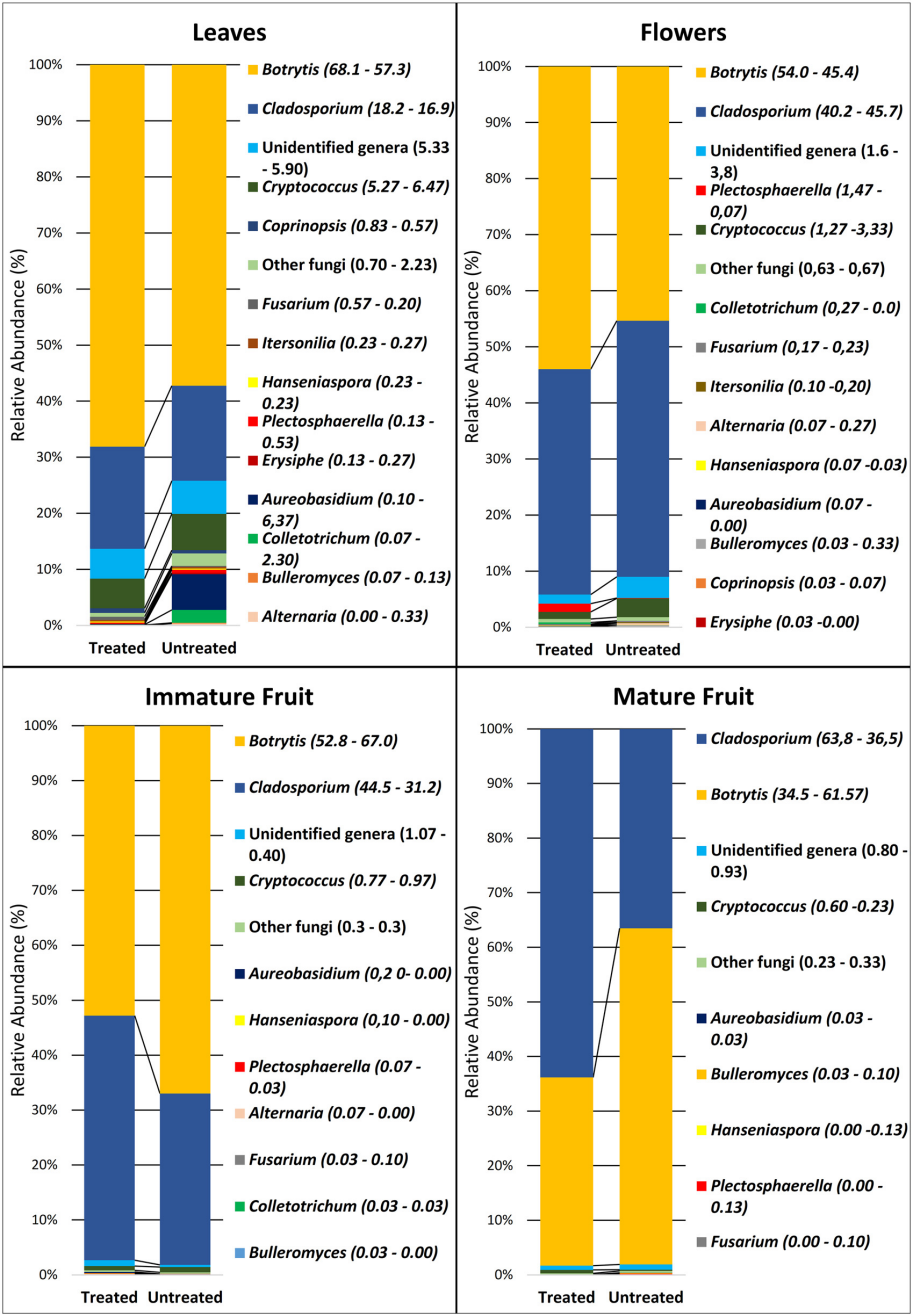
Relative abundance of the fungal genera in treated and untreated strawberry flowers, leaves and immature and mature fruit. The exact relative abundance (percentage) of each genus in treated (left number) and untreated (right number) samples is reported between parentheses.

The most abundantly detected genera were *Botrytis* spp. and *Cladosporium* spp. with the percentage of reads ranging from 70% in leaves to 99% in fruit. *Botrytis*, represented by 12 different sequence types (STs), was the most abundant genus with a RA of 53.8% (Fig 6). According to the phylogenetic analysis, all STs clustered with reference sequences of *B*. *cinerea* and other closely-related species of the same genus having identical or very similar ITS2 sequences (S1 File). The resulting clustering of sequences did not enable the identification of STs at the level of species but confirmed the identification of the genus as *Botrytis* since sequences were clearly separated from those of the related genera *Monilinia* and *Sclerotinia*.

The genus *Cladosporium* had a RA of 37.80% and was most abundant on treated fruit (Fig 6). The phylogenetic analysis of its sequences enabled the identification of 29 different STs that were related to the *Cladosporium pullulans* complex (19 STs) and to the species *Cladosporium sphaerospermum* (10 STs). STs related to the *C*. *pullulans* complex were much more abundant, representing 97.2% of the total *Cladosporium* spp. reads. The application of fungicides caused a significant reduction in the RA of *Botrytis spp*. and an increase in the RA of *Cladosporium* spp. on mature and immature fruit (Fig 6). This trend, however, was not evident in flowers or leaves (Fig 6).

*Cryptococcus* spp. was the third most abundant genus detected. It was less abundant on mature and immature fruit and most abundant on flowers and leaves (Fig 6). Fungicide treatments reduced the RA of *Cryptococcus* spp. on leaves from 6.47% to 5.27% and from 3.33% to 1.27% on flowers. The phylogenetic analysis of STs of this genus revealed a high genetic diversity with the identification of six different species (*C*. *victoriae*, *C*. *tephrensis*, *C*. *heimaeyensis*, *C*. *oeirensis*, *C*. *stepposus*, *and C*. *flavescens*) and two phylotypes clustering within the genus but not related to currently known species (S1 File).

Other genera represented a limited portion of the detected fungal populations. The genus *Aureobasidium*, represented by 3 STs, all identified as *A*. *pullulans*, was most abundant on leaves and less abundant on other organs. The leaf population of *A*. *pullulans* was significantly influenced by the fungicide treatments as evidenced by its RA on untreated leaves (6.37%) vs. treated leaves (0.1%).

Other taxa detected in the investigated organs included *Plectosphaerella cucumerina*, two different phylotypes of *Fusarium* spp. closely related to *F*. *avenaceum* and *F*. *equisety*, *Hanseniaspora uvarum*, and *Bulleromyces albus* (Fig 6; S1 File). Sequences related to *Alternaria Sect*. *Alternata* (a single ST) and sequences related to the genus *Colletotrichum*, identified as *C*. *acutatum s*.*str*. (a single ST) and *C*. *lini* or *C*. *americae-borealis* (two STs), were detected in flowers, immature fruit, and leaves. Finally, *Coprinopsis cinerea* (a single ST), *Itersonia perplexans* (two STs) and a ST clustering within the *Erysiphe aquilegiae* clade, were detected in flower and leaf samples (Fig 6; S1 File).

Other fungal genera with a very low RA cumulatively represented a RA ranging from 0.30% (immature fruit) to 2.23% (untreated leaves). The incidence of these fungi decreased to 0.7% in treated leaves. Unidentified fungal genera had a cumulative RA ranging from 0.4% (untreated immature fruit) to 5.9% (untreated leaves). Lastly, fungal genera representing known strawberry pathogens, including *Verticillium*, *Penicillium*, *Aspergillus*, *Sclerotinia*, *Phoma*, *Rhizopus*, *Ceratobasidium*, *Mycosphaerella* and *Septoria* were detected with a RA ≤0.1%.

## Discussion

Results of the present study indicate that a wide variety of fungal taxa inhabit strawberry plants. Some of them are known to be resident while others have not been previously reported on this host plant. The study also demonstrated that the composition and diversity of fungal communities, as evidenced by their alpha and beta diversity, vary significantly on different plant organs (leaf, flower, and mature and immature fruit). The alpha diversity analysis indicated that leaves presented the most diverse fungal community, followed by flowers. This ranking of diversity is in agreement with a previous study conducted on olive trees, in which the fungal community of the same organs was analyzed [9]. In that study, it was suggested that the greater diversity present on leaves may be due to the higher surface/volume ratio of leaves relative to fruit. Additionally, leaves do not change as much as fruit in shape, color, and nutrient composition as they develop and mature. Leaves also have a longer life than flowers that quickly develop into fruit.

The fungal population associated with strawberries was characterized by the prevalence of two genera, *Botrytis* and *Cladosporium*, while other fungi exhibited a very low RA. The prevalence of *Botrytis* spp. over other fungal genera was somewhat unexpected considering its low frequency of isolation in previous studies which have investigated strawberry fungal populations using conventional cultural methods based on the nutritive media plating of wash water from fruit [30]. Sylla et al. (2013), using 454 pyrosequencing, indicated that while *Botrytis* was not detected initially, its RA increased to as high as 37% in subsequent samplings and its presence was unaffected by several applications of a variety of biocontrol agents [6]. The high RA of *Botrytis* spp. detected in the present study in asymptomatic leaves, flowers, and mature and immature fruit may be partially explained by the high incidence of grey mold infections occurring in the investigated field during the sampling period (Schena L., personal communication). These infections may have served as a source for the contamination of healthy tissues with *B*. *cinerea* conidia. Furthermore, *B*. *cinerea* readily colonizes floral tissues and establishes latent infections which would be difficult to detect by plating wash water from fruit because the infections are internal and the fungus is dormant [5,31]. On the other hand, the heterokaryotic nature of *B*. *cinerea*, which can possess an average of 3–6 nuclei per cell [32], may contribute to overestimating the fungal population using molecular detection methods. Although, *Botrytis* was the main target organism of the chemical treatments that were applied, the RA of *Botrytis* on leaves was unaffected by their application, though the presence of *Botrytis* was reduced in both immature and mature fruit. These results suggest that there is a large potential reservoir of *Botrytis* conidia that at any point in time could serve as source of inoculum for infecting fruit.

The high prevalence of two genera, *Botrytis* and *Cladosporium*, over all other detected fungi may be related to the intensive cultivation system used in the investigated field and the use of fungicides. Chemical applications have been reported to have a significant impact on non-target organisms and reduce overall genetic diversity [8,33]. Indeed, the phyllosphere population characterized in the present study is quite different compared to that determined in a previous investigation focusing on organically grown strawberries, where several fungal genera had a very high RA [34]. Supporting this previous study, beta diversity analyses in the current study revealed that exposure of strawberries to chemical treatments resulted in a significant reduction on the fungal composition of leaves and flowers. In the case of immature and mature fruit, significant differences in fungal diversity were only observed using weighted metrics suggesting that fungicide applications did have an effect on the RA of specific fungal taxa but not on the overall composition of the fungal community. The overall higher fungal diversity detected in leaves and flowers, relative to fruits may have reflected different responses to the applied chemicals. It is important to note that the present study was conducted in a strawberry field on a farm using conventional practices and that the untreated plants did not receive the conventionally-used fungicidal treatments for only a month. This fact underscores the ability of fungal communities to change significantly over such a short period of time.

A more in-depth analysis of the DNA sequences of the identified OTUs provided additional information at the genus and species level [9]. The analysis of sequences related to *Botrytis* spp. was problematic and made their identification at the species level difficult. This was due to the existence of several accepted species with very similar or identical ITS sequences [35]. Considering the relevance of grey mold to strawberries it can be assumed that the majority of the detected sequences belong to *B*. *cinerea*, even though the presence of other species cannot be excluded. The detection of 12 distinct STs suggests that there is a great deal of diversity which perhaps is a reflection of the wide distribution and host range of *Botrytis* species [36]. Most of this genetic diversity is likely to remain undetected using traditional culturing methods since, in the present study, a single ST was predominant above other STs of *Botrytis* and represented 83.8% of the total sequences obtained for this genus. However, the heterokaryotic nature of *B*. *cinerea* should also be considered as potentially contributing to the detected level of molecular diversity [32].

The genus, *Cryptococcus*, was the most abundant yeast identified on the examined strawberry organs in the present study, being represented by six species and two non-identified taxa. The abundance of *Cryoptococcus* was affected by the application of the chemical treatments and was significantly reduced in both leaves and flowers. This finding is in agreement with the high RA of this genus found in the phyllosphere of organic strawberries, where it was the most abundant fungus [34]. Interestingly, *Cryoptococcus* has often been isolated from fruit washings and has been identified as a biocontrol agent for the management of postharvest diseases [37]. Due to its potential role as an effective biocontrol agent, more studies should be conducted on its role in inhibiting plant pathogens in natural plant microbial communities. Perhaps methods could be found to support and/or enhance its presence. The yeast-like fungus, *A*. *pullulans*, has also been isolated from plant washings and used to control postharvest diseases on a number of different commodities [30,38,39]. The overall detected population of *A*. *pullulans* in the present study was lower than expected considering previous studies that focused on the phylloplane of strawberry and other plant species [9,34]. In the current study, a high population of *A*. *pullulans* was only detected in untreated leaves (6.37%). However, *A*. *pullulans* was significantly impacted by the fungicide application, being reduced in abundance down to 0.1% in treated leaves. As suggested for *Cryptococcus*, the use of chemicals in the strawberry production system examined in the present study may be responsible for the overall low abundance of *A*. *pullulans* and indicate the need for further studies to better understand the role of *A*. *pullulans* in strawberry fungal communities, as well as its potential use to prevent the establishment of infections by plant pathogens.

The detection of *Colletotrichum* species was expected considering their major role as causal agents of strawberry anthracnose. Surprisingly, among the several *Colletotrichum* species responsible for strawberry anthracnose [40,41], only *C*. *acutatum s*. *str*. was detected and at a very low percentage. On the other hand, molecular analyses revealed the presence of sequences associated with two different *Colletotrichum* species (*C*. *lini* and *C*. *americae-borealis*) having identical ITS sequences [42]. Both species have been reported on flax and several other hosts but not on strawberry [42]. A single ST related to *Alternaria Sect*. *Alternaria* was detected with a low RA in flowers, immature fruit, and leaves. The genus *Alternaria* was found to be quite abundant in the phyllosphere of organic strawberries [34] and in the carposphere of conventional and organic strawberries [5]. Jensen (2013) hypothesized that this fungus may have a role as a mycotoxin producer in strawberries. Two different phylotypes of *Fusarium* spp. were detected in the present study and were found to be genetically related to *F*. *equiseti* and *F*. *avenaceum*, although the available data on genetic diversity within the ITS2 region did not enable the unequivocal identification of these two species. *Fusarium equiseti* has been reported to cause damping-off and root rot in cucurbits [43] while *F*. *avenaceum* is a cosmopolitan plant pathogen with a wide host range [44]. The role of these microorganisms in the strawberry phylloplane is unknown, but the potential production of mycotoxins cannot be completely excluded. Our study also revealed the presence of the biotrophic pathogen, *Erysiphe aquilegiae*, on leaves and flowers. This fungus is the causal agent of powdery mildew in species of the family *Ranunculaceae* [45] and in *Catharanthus roseus* [46] but has never been associated with strawberries. Surprisingly, *Sphaerotheca macularis f*. *sp*. *fragariae*, the causal agent of strawberry powdery mildew [47] was not detected. Among the other detected fungal species, *P*. *cucumerina* is a known pathogen of melon and the causal agent of fruit and collar rot in several plant species but not on strawberry [48]. *Itersonilia perplexans* is the causal agent of foliar and petal blight on several cut-flowers [49]. *Hanseniospora uvarum* has been isolated from the surface of fruit such as grapes and is known for its role in natural fermentation and for the secretion of a killer toxin against *Saccharomyces cerevisiae* [50]. Important strawberry pathogens such as *Rhizoctonia*, *Cercospora* and *Macrophomina* were not detected.

In conclusion, the results of the present study indicate that strawberry plants support a large diversity of fungal organisms, although two genera, *Botrytis* and *Cladosporium*, were more abundant than all of the other identified genera combined, representing 70–99% of the RA. Several fungal species that are pathogenic on other plant species, and that would not be intuitively expected to be present on strawberry plants, were among the detected microorganisms while some common strawberry pathogens were less evident or not detected. The study also revealed a high degree of diversity in the ITS sequence within certain taxonomic groups. Both these findings raise significant questions about the natural fungal community present on plants and about our classification of plant pathogens, and fungal species in general. One could raise the question whether or not some taxonomic units are correctly assigned or alternatively if our understanding of the genetic identity of an infectious agent is comprehensive enough to account for some level of genetic diversity within a species. These questions are highlighted in the present study by the genetic diversity observed within *Botrytis*, and the genetic (sequence-based) presence of species that are pathogenic on hosts other than strawberry, while there was a failure to identify the strawberry-equivalents of the same disease-causing organisms. These questions will only be resolved as more metagenomic data about natural microbial populations on plants accumulates and our understanding of genetic diversity within microbial species increases.

## Supporting Information

S1 File Molecular identification of detected sequence phylotypes. Phylogenetic trees were built using unique sequences representative of sequence types (STs) of the most relevant fungal genera detected in the present study and validated reference sequences of each fungal genus. Numbers in parentheses along with STs (MIDs) indicate the percentage of sequences represented by each ST within each genus. Numbers on nodes represent the posterior probabilities for the maximum likelihood method.
(DOCX)
