# Supplementary material for: Metagenomic Analysis of Fungal Diversity on Strawberry Plants and the Effect of Management Practices on the Fungal Community Structure of Aerial Organs
Source: PLoS One. 2016 Aug 4;11(8):e0160470. doi: 10.1371/journal.pone.0160470 (PMC4973904; doi:10.1371/journal.pone.0160470)
Supplement: S1 File — Phylogenetic trees were built using unique sequences representative of sequence types (STs) of the most relevant fungal genera detected in the present study and validated reference sequences of each fungal genus. Numbers in parentheses along with STs (MIDs) indicate the percentage of sequences represented by each ST within each genus. Numbers on nodes represent the posterior probabilities for the maximum likelihood method. (DOCX) [file pone.0160470.s001.docx]

**S1_File. Phylogenetic identification of detected sequence types (STs).** Trees were built using unique sequences representative of STs of the most relevant fungal genera detected in the present study and validated reference sequences of each fungal genus. Numbers in parentheses along with representative STs (MIDs) indicate the percentage of sequences represented by each ST within each genus. Numbers on nodes represent the posterior probabilities for the maximum likelihood method.
